# Immediate effects of the phonation into a straw exercise

**DOI:** 10.1590/S1808-86942011000400009

**Published:** 2015-10-19

**Authors:** Claudia Barsanelli Costa, Luis Henrique Chechinato Costa, Gisele Oliveira, Mara Behlau

**Affiliations:** 1 Specialization - Centro de Estudos da Voz (Voice Study Center), clinical speech therapist; 2 Doctoral degree in medicine, Faculdade de Ciências Médicas Santa Casa (Santa Casa Medical Science School), SP. Professor Auxiliar da Faculdade de Medicina de Jundiaí; 3 Doctoral degree in speech therapy, Universidade Federal de São Paulo (São Paulo Federal University). Professor and vice-coordinator of the Centro de Estudos da Voz (Voice Study Center); 4 Doctoral degree in human communication disorders, Universidade Federal de São Paulo (São Paulo Federal University). Director of the Centro de Estudos da Voz (Voice Study Center). Centro de Estudos da Voz - CEV (Voice Study Center)

**Keywords:** speech acoustics, voice, voice training

## Abstract

**Abstract:**

Immediate effects of an exercise with the vocal tract semi-closed.

**Objective:**

To investigate the immediate effects of the phonation exercise in a straw in individuals with and without vocal fold lesions.

**Materials and Methods:**

Prospective study. 48 individuals, aged between 18 and 55 years participated in the study, and they were distributed into two groups: GL- with a benign vocal fold lesion; GSL-without a lesion. The following analyses were carried out before and after the straw phonation exercises: voice self-analysis, [ε] vowel auditory-perception analysis, selected parameters acoustics (VoxMetria 2.6) and videolaryngoscopy.

**Results:**

The vocal self assessment indicated a statistically significant improvement on voice emission in GL individuals (*p*=0.015). There were no statistically significant differences before and after the exercise and between the groups, in the videolaryngoscopy, acoustic and auditory-perception assessments.

**Conclusion:**

The straw phonation exercise caused positive effects, seen upon voice self-assessment, indicating an easier and better voice upon phonation.

## INTRODUCTION

Voice training may be employed to prevent dysphonia or to rehabilitate patients with dysphonia. Selection of exercises should be customized and based on therapeutic testing; the goal is to attain voice emission with minimal effort and maximal efficiency. Semi-occluded vocal tract exercises (SOVTE) have been widely used in clinical practice to improve voice performance. In spite of variations in the technique, occlusion is typically between the anterior portion of the mouth and the lips.

Phonation into a straw is one of the variations of SOVTE^1^, a voice reeducation technique since it optimizes the facial vibration sensations resulting from increased intraoral acoustic pressure. This approach allows individuals to adjust their vocal tract optimally to improve voice performance^2^.

Phonation with semiocclusion alters the acoustic impedance of the vocal tract, which is usually lower than that of the glottis, and generates a positive pressure. In principle, acoustic impedance of the vocal tract may change voice function in two levels: acoustic-dynamic interaction, and mechanical-acoustic interaction - the interactions between pressures in the vocal tract and vibration of the vocal folds. Partial constriction of the vocal tract with a straw is accompanied by a reactive type inertance that facilitates self maintenance of vocal fold vibrations by reducing the phonation pressure threshold^3^, ^4^, ^5^, ^6^.

The literature contains a few studies that have investigated the effects of phonation with SOVTE, such as changes in the fundamental frequency (F_0_) and positive changes with improved voice performance, resulting in utterances with minimal effort and maximal efficiency. Studies of subjects with vocal fold lesions, after many hours of using speech daily and complaints of voice fatigue, have yielded positive results of phonation effects with SOVTE^1^,^4^,^7^.

The purpose of this study was to investigate the short-term effects of phonation exercises with straws in subjects with and without vocal fold lesions.

## METHODS

The institutional review board approved this study (no. 715/05). Participants signed a free informed consent form, as required by the Resolution 196/96 - CNS.

The study sample consisted of 48 subjects (40 female and 8 male) aged from 18 to 55 years, divided into two groups: one with laryngeal lesions (GL), and the other without laryngeal lesions (GSL). The GL group comprised 23 subjects (20 female and 3 male) aged from 20 to 50 years (mean - 35.5 years) with benign vocal fold lesions (GL) - nodules, cysts, polyps, and Reinke's edema. The GSL group comprised 25 subjects (20 female and 5 male) aged from 21 to 55 years (mean - 37 years), with no lesions (GSL).

The exclusion criteria were as follows: systemic diseases that impact voice, and diagnosed psychiatric or neurologic conditions. Participants in the group with complaints about voice were recruited from an otorhinolaryngology and speech therapy clinic in the city of Jundiaí, who had sought the clinic because of voice related complaints. The control group consisted of volunteers with no voice complaints and no visible lesion in routine exams (GSL).

The following procedures were carried out to assess voice and the larynx before and after the phonation exercises into a straw with a defined time: self assessment of voice, perceptual auditory evaluation, acoustic analysis, and laryngoscopy.

**Figure 1:**
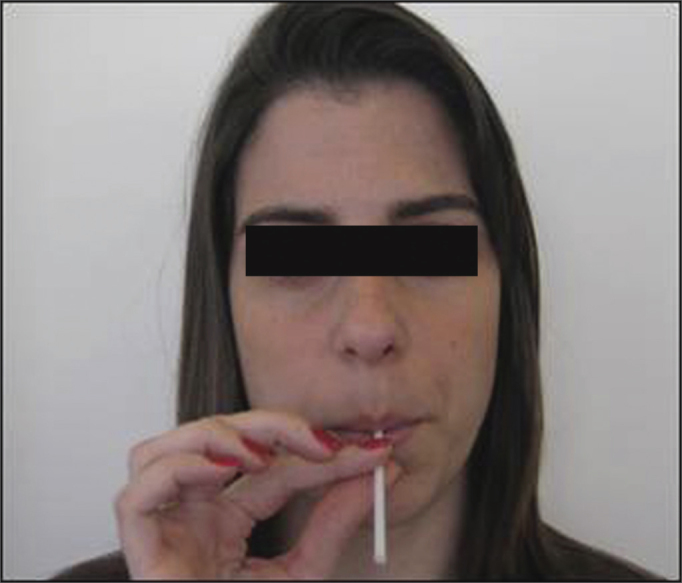
Semi-occluded vocal tract exercises, phonation into a straw.

Patients were instructed to perform phonation exercises into a straw, which is one of the modalities of the SOVTE. A rigid plastic straw 8.7 cm long and 1.5 mm in diameter was used in this exercise. After the examiner demonstrated the exercise, participants in turn repeatedly uttered an undifferentiated sound similar to a prolonged “vu” during one minute at a self-selected mid-range frequency and intensity, holding the straw between their teeth and closing their lips so that airflow passed continuously through the straw (Fig. 1).

Each participant assessed the impact of the exercise on voice immediately afterwards by answering the following voice self-assessment protocol: easier voice, better voice, easier and better voice, or no difference after the exercise.

A Le Son model MP-68 unidirectional microphone placed at a fixed distance (3 cm) and at a 45 degree directional pickup angle to the mouth of speakers was used to record speech for the perceptual auditory and acoustic evaluations; this procedure was done in a silent room. Voices were recorded directly on a Sony Vaio computer running the FonoView (CTS informatics, 2.6) acoustic evaluation software. Voices of participants were randomly (pre- and post-exercise) edited in pairs. The beginning and the end of each utterance were discarded to avoid effects on the analysis due to the unstable conditions of these segments.

Two speech therapists, both experts on voice with at least three years experience and who did not know the subjects or pre- and post-exercises beforehand, individually carried out the perceptual auditory analysis. The instruction was to compare two segments uttered by the same subjects and to mark the best segment or absence of differences between segments. Ten percent of the samples were repeated to verify intra-evaluator reliability in both analyses.

F_0_, jitter, shimmer were selected as parameters in the acoustic analysis; segments before and after the exercises were used. The software Voxmetria (CTS informatics) was applied using extracts of the uttered sustained vowel.

Laryngoscopy was done with a Storz 10 mm rigid laryngoscope coupled to a Toshiba camera model IK-CU44A; images were recorded on a DVD. One otorhinolaryngologist examined the larynxes of subjects, without topical anesthesia, immediately before and after the exercises. Patients were asked to utter the vowel [ε] in a prolonged manner with their habitual voice.

Two parameters were selected for evaluating the laryngeal configuration; two segments per subject were compared (recording moment not identified): constriction of the vestibule, and presence of a gap between vocal fold. The same evaluators that did auditory testing looked for these parameters. Ten percent of the sample was tested again to check for intra-evaluator reliability in both analyses.

The results were analyzed statistically; the significance level was 0.05 (5%). Pearson's chi square test was applied to check for possible differences between the study groups (voice self-assessment), between testers in the perceptual auditory assessment (intra- and inter-evaluator reliability), and in videolaparoscopy. The Wilcoxon signed rank test was applied in the acoustic analysis to check for possible differences in both testing moments per gender and per study group.

## RESULTS

Table 1 shows the voice self-assessment values after phonation with straw SOVTE, using Pearson's chi square test (*p*=0.015).

**Table 1:** Voice self-assessment after SOVTE - phonation into a straw

Group	Easier voice	Better voice	Easier and better voice	No difference
GL	6 (26.09%)	5 (21.74%)	8 (34.78%)	4 (17.39%)
GSL	7 (28.00%)	2 (8.00%)	2 (8.00%)	14 (56.00%)

Significance level a=0.05. Pearson's chi-square test

Sample = 23 (group with lesion) 25 (group without lesion)

Table 2 shows the response frequency in the perceptual auditory assessment to the best utterance of vowels before and after exercise. Pearson's chi square test was applied here (*p*=0.998).

**Table 2:** Response frequency in the perceptual auditory analysis - best utterance of vowels, before and after exercises

Group	Pre better	Post better	Unaltered
GL	13 (28.26%)	14 (30.43%)	19 (41.30%)
GSL	14 (28.00%)	15 (30.00%)	21 (42.00%)

Significance level a=0.05. Pearson's chi-square test

Sample = 23 (group with lesion) 25 (group without lesion)

Tables 3 and 4 show the mean fundamental frequency (F_0_, in Hz) of the vowel [ε], as well as jitter e shimmer, before and after phonation into a straw exercises according to each group (with or without lesion) and each sex. The Wilcoxon signed rank test was applied to reach the statistical results.

**Table 3:** Mean fundamental frequency (F_0</sb>_ in Hz), of the vowel [ε], and of jitter and shimmer, before and after phonation into a straw exercises, according to the group with lesions (GL) and gender

	GL					
		MALE			FEMALE	
	PRE	POST	(*p*)	PRE	POST	(*p*)
F_0_ MEAN	157.01	131.96	0.109	206.91	211.24	0.263
JITTER	1.52	1.91	0.593	0.77	0.77	0.888
SHIMMER	9.57	10.12	0.999	9.47	6.74	0.629

Significance level a=0.05. Wilcoxon signed rank test

Sample = 23 (group with lesion) 25 (group without lesion)

**Table 4:** Mean fundamental frequency (F_0_ in Hz), of the vowel [ε], e of jitter and shimmer, before and after phonation into a straw exercises, according to the group without lesions (GSL) and gender.

	GSL					
		MALE			FEMALE	
	PRE	POST	(*p*)	PRE	POST	(*p*)
F0 MEAN	122.83	125.83	0.686	199.9	196.03	0.433
JITTER	0.17	0.25	0.345	0.47	0.43	0.779
SHIMMER	2.95	3.53	0.500	4.31	4.45	0.751

Significance level a=0.05. Wilcoxon signed rank test

Sample = 23 (group with lesion) 25 (group without lesion)

Table 5 shows videolaryngoscopy for analyzing the parameters constriction of the vestibule and presence of a gap between vocal fold before and after phonation into a straw exercises. Pearson's chi square test was applied to reach the statistical results.

**Table 5:** Videolaryngoscopy of the parameters: constriction of the vestibule and glottic gap, before and after phonation into a straw exercises.

Parameters	Group	Pre better	Post better	Unaltered	*p*
Constriction of the vestibule	GL	7 (15.22%)	7 (15.22%)	32 (69.57%)	0.895
	GSL	8 (16.33%)	9 (18.37%)	32 (65.31%)	
Presence glottic gap	GL	2 (8.00%)	4 (16.00%)	19 (76,.00%)	0.404
	GSL	1 (2.04%)	6 (12.24%)	42 (85.71%)	

Significance level a=0.05. Pearson's chi-square test.

Sample = 23 (group with lesion) 25 (group without lesion)

Self-assessment of voice yielded more positive than negative effects, which were statistically significant (*p*=0.015) - the best results were found in the group with vocal fold lesions (Table 1).

Table 2 shows the perceptual auditory assessment, where the results of two exercises did not differ statistically. Intra-evaluator reliability was higher than 90% in all conditions. Inter-evaluator agreement was about 56% in all tasks.

Acoustic analysis (Tables 3 and 4) revealed no statistically significant differences among groups and gender, of phonation into a straw exercises.

Analysis of the laryngeal configuration (Table 5) revealed no statistically significant difference in constriction of the vestibule relative to groups and sex. GL: (pre=15.22%, post=15.22%, *p*=0.895); GSL (pre=16.30%, post=18.37%, *p*=0.895), and glottic gap. GL: (pre= 8%, post=16%, *p*= 0.404). Intra-evaluator reliability was higher than 90% in all conditions. Inter-evaluator agreement was about 53% in all tasks.

## DISCUSSION

The vocal tract operates based on the laws of physics; the interaction between air flow and muscle control result in continued vocal fold oscillations that produce sound. Voice economy is attained in the balance between these relations.

The vocal tract affects vocal fold oscillations. The effect of SOVTE in increasing supraglottic pressure is well known - a retroflex resonance effect occurs in the semioccluded vocal tract. As glottic production remains active, the full area of the vocal tract expands, and increased pressure in the vocal tract raises the sound sensation in the mouth. The latter results in production of sound, which facilitates voice training.

Computerized models have been used in simulations of the vocal tract and the effects of therapy on several variables; this indicates that there is increased vocal tract inertance. The effects on clinical practice remain relatively unknown, which justifies studies such as this.

In the present study, both groups reported benefits with phonation exercises into a straw, in the voice self-assessment (Table 1). There were more positive results in the GL than in the GSL (*p* 0.015), indicating that phonation exercises into a straw could be included in voice training, voice therapy, and voice warm-up programs^8^, ^9^, ^10^. Subjects are expected to perceive how to produce voice economically, using it is common speech and singing - in voice producing conditions that differ from those during exercises. Partial lip occlusion and vocal tract lengthening could be used in emphasis and tone training^8^. Sampaio et al. (2008) also found similar results in a study with speech therapists^11^.

There was no statistically significant difference between groups in the perceptual auditory assessment, or before and after exercises. This result differed from those in other studies that reported improved voice quality and stability^11^, ^12^, ^13^. Published studies in the literature in general have shown that SOVTE improve voice in perceptual auditory assessments.

An inter-evaluator agreement around 56% in the perceptual auditory assessment (Table 2) was found because only indication of the best utterance was asked for, and not any characterization of specific parameters. A further evaluation using specific parameters may help understand the aspects involved in these results.

Acoustic analysis parameters (Tables 3 and 4) varied discretely, and were not significant. A few studies of dysphonic voices have suggested that an altered F_0_ may be related with decreased stress, vocal tract adjustments, and reactance^9^,^11^,^14^. Stress during testing may elevate the larynx. The crichothyroid muscle is activated during exercise, which is important for controlling frequency; its contraction raises the F_0_^10^. Analyses of the delayed effects of exercise may indicate more evident changes in the selected acoustic parameters. Story, Laukkanen, and Titze^4^ have investigated that effects of constriction, vocal tract lengthening, and acoustic impedance in seven computerized vocal tract configurations; these authors found increased impedance and F_0_ lowering in a configuration that corresponds to phonation into a straw, relative to the labial fricative.

Visual evaluation of the larynx (Table 5) yielded no statistically significant difference, possibly because execution time was insufficient; furthermore, the GL was rather heterogeneous, as there were different types of laryngeal lesions. Nascimento (2005) found similar results^13^. Further studies comparing laryngeal constriction and the presence or absence of a gap according to the number of exercises, controlling the types of vocal fold lesions, may clarify this issue.

An inter-evaluator agreement around 53% in the visual evaluation of the larynx (Table 5) occurred because no prior training was given and specific parameters were not asked for. A study taking into account specific parameters may help understand these aspects of the results.

Based on clinical observation, the fact that there were no statistically significant results - except in self-assessments - was probably because the effect of phonation into a straw exercises appears to be cumulative and long term, rather than short term. It is possible that the duration of the exercise (1 minute) or the selected evaluation parameters were not sensitive enough for more evident results. Further studies of the duration of exercises may clarify this issue.

Our results may be useful to understand the effects of phonation into a straw, and may guide its application in different conditions.

## CONCLUSION

Phonation into a straw exercises yielded positive results, verified mainly in voice self-assessments, with reported easier and better voice utterance.
